# Quantitative screening of serum protein biomarkers by reverse phase protein arrays

**DOI:** 10.18632/oncotarget.25976

**Published:** 2018-08-24

**Authors:** Zhizhou Kuang, Ruochun Huang, Zhimin Yang, Zhiqiang Lv, Xinyan Chen, Fuping Xu, Yu-Hua Yi, Jian Wu, Ruo-Pan Huang

**Affiliations:** ^1^ RayBiotech Inc, Guangzhou, China; ^2^ RayBiotech Inc, Parkway Lane, Norcross, GA, USA; ^3^ Guangdong Provincial Academy of Chinese Medical Sciences, Guangzhou, China; ^4^ Guangdong Provincial Hospital of Chinese Medicine, Guangzhou, China; ^5^ The Third Affiliated Hospital of Sun Yat-Sen University, Guangzhou, China; ^6^ South China Biochip Research Center, Guangzhou, China

**Keywords:** reverse phase protein array, biomarker screening, serum protein, hepatocellular carcinoma

## Abstract

Screening biomarkers in serum samples for different diseases has always been of great interest because it presents an early, reliable, and, most importantly, noninvasive means of diagnosis and prognosis. Reverse phase protein arrays (RPPAs) are a high-throughput platform that can measure single or limited sets of proteins from thousands of patients' samples in parallel. They have been widely used for detection of signaling molecules involved in diseases, especially cancers, and related regulation pathways in cell lysates. However, this approach has been difficult to adapt to serum samples. Previously, we developed a sensitive method called the enhanced protein array to quantitatively measure serum protein levels from large numbers of patient samples. Here, we further refine the technology on several fronts: 1. simplifying the experimental procedure; 2. optimizing multiple parameters to make the assay more robust, including the support matrix, signal reporting method, background control, and antibody validation; and 3. establishing a method for more accurate quantification. Using this technology, we quantitatively measured the expression levels of 10 proteins: alpha-fetoprotein (AFP), beta 2 microglobulin (B2M), Carcinoma Antigen 15-3(CA15-3), Carcinoembryonic antigen (CEA), golgi protein 73 (GP73), Growth differentiation factor 15 (GDF15), Human Epididymis Protein 4 (HE4), Insulin Like Growth Factor Binding Protein 2 (IGFBP2), osteopontin (OPN) and Beta-type platelet-derived growth factor receptor (PDGFRB) from serum samples of 132 hepatocellular carcinoma (HCC) patients and 78 healthy volunteers. We found that 6 protein expression levels are significantly increased in HCC patients. Statistical and bioinformatical analysis has revealed decent accuracy rates of individual proteins, ranging from 0.617 (B2M) to 0.908 (AFP) as diagnostic biomarkers to distinguish HCC from healthy controls. The combination of these 6 proteins as a specific HCC signature yielded a higher accuracy of 0.923 using linear discriminant analysis (LDA), logistic regression (LR), random forest (RF) and support vector machine (SVM) predictive model analyses. Our work reveals promise for using reverse phase protein arrays for biomarker discovery and validation in serum samples.

## INTRODUCTION

Biomarkers are key indicators used for early, rapid and accurate diagnosis for most diseases. With proper biomarkers, the individual therapeutic feedback against these diseases could be evaluated promptly. Newly discovered biomarkers could also provide novel targets for drug design. While gene sequencing technology has generated an unprecedented amount of genetic information about disease status [1–3], and determined groups of nucleic acid biomarkers such as SNPs and even microRNAs, proteins provide a rich potential biomarker pool for disease identification because diseases are a reflection of the deregulation of protein products and protein networks that respond to and/or result from external and internal stimuli [4–6]. In addition, changes in protein structure such as modifications and misfolding have also been shown to be crucial in development and progression of some diseases, which cannot be ascertained from gene sequence assays.

For identification of protein biomarkers, high-throughput screening platforms have shown powerful technical advantages, differing from traditional approaches such as ELISA and western blot. Currently, two technologies including antibody arrays and reverse phase protein arrays (RPPAs) have been wildly employed in the discovery and validation of biomarkers and have produced promising results for many diseases including cancers, cardiovascular diseases and neurodegeneration diseases [7]. Antibody array technology facilitates the detection of hundreds of targets simultaneously from samples with a similar sensitivity and specificity as ELISA. RPPA technology, which was adopted from dot-blot technology, measures protein expression levels in thousands of samples simultaneously through the arraying of microspots of protein samples on a solid matrix and probing with highly specific antibodies. RPPA is a cost–effective and robust platform offering a high-throughput approach to screen biomarkers or validate candidate markers with a tiny amount of sample over a huge population of samples. This is ideal for projects requiring observation over time, before and after treatment, between disease and non-disease states as well as between responders and nonresponders, etc. Since the first publication in 2001 [8], RPPAs have been successfully applied in monitoring epigenetic changes of proteins, such as phosphorylation, involved in disease-related regulation pathways in tissue or cell lysate samples, especially in cancers [9–10].

Because blood biomarkers offer an early, reliable, and, most importantly, noninvasive means of diagnosis and prognosis, screening biomarkers in serum samples for different diseases has always been of great interest. While RPPAs have been widely applied in the detection of potential biomarkers in tissue samples and cell lysates, it has rarely been reported to be applied in serum protein investigations as a high-throughput tool for the most easily obtained clinical samples. Previously, we developed a sensitive method called the enhanced protein array to quantitatively measure serum protein levels from large numbers of patient samples. This method is similar to the RPPA system, but additional steps to increase the assay sensitivity through coating corresponding target antibodies on membrane arrays were included [11]. In this study, to further refine this high-throughput technology, we have developed a reliable RPPA system for serum sample detection by 1. simplifying the experimental procedure; 2. optimizing multiple parameters to make the assay more robust, including the support matrix, signal reporting method, background control, and antibody validation; and 3. establishing a method for more accurate quantification.

Hepatocellular carcinoma (HCC) is one of the most common malignant tumors with a high rate of morbidity and mortality in the world, affecting approximately one million individuals annually worldwide. Because most of patients with HCC are diagnosed at a late stage, the prognosis of HCC patients is generally very poor with a 5-year survival rate around 10% [12]. Therefore, early diagnosis is crucial for improving the survival rate of HCC patients. Currently α-fetoprotein (AFP) combining with pathological detection are commonly used in the early diagnosis of liver cancer. However, the specificity and sensitivity of AFP are very limited [13]. More recently, with the development of molecular biology, more and more serum proteins have been identified, including proteantigens, cytokines and enzymes, displaying the potential association with the diagnosis and prognosis of liver cancer. Undoubtedly more new tumor markers are required for effective early diagnosis. By using our optimized RPPA system in this study, we have chosen to detect several serum protein targets including three groups: 1). AFP and GP73, which are commonly considered to be highly related with HCC; 2). B2M, CEA, GDF15, IGFBP2, OPN and PDGF-Rb, which are uncertain about the association with liver cancer but have involvement in liver diseases or multiple cancers; 3). CA15-3 and HE4, which have been applied as other tumor cancer biomarkers but not HCC. With this technology, we have successfully quantitatively measured the protein expression levels from serum samples of hepatocellular carcinoma (HCC) patients and healthy controls and found differential expression in 6 detected proteins between HCC patients and the healthy controls.

## RESULTS

### Establishment of the RPPA reaction system

To establish the RPPA with increased accuracy and efficiency in the simplest way to assay protein targets in serum samples, we have optimized the reaction system in multiple ways, including the support matrix, antibody labeling combination and selection, background reduction, colorimetric method and sample preparation.

**Figure 1:**
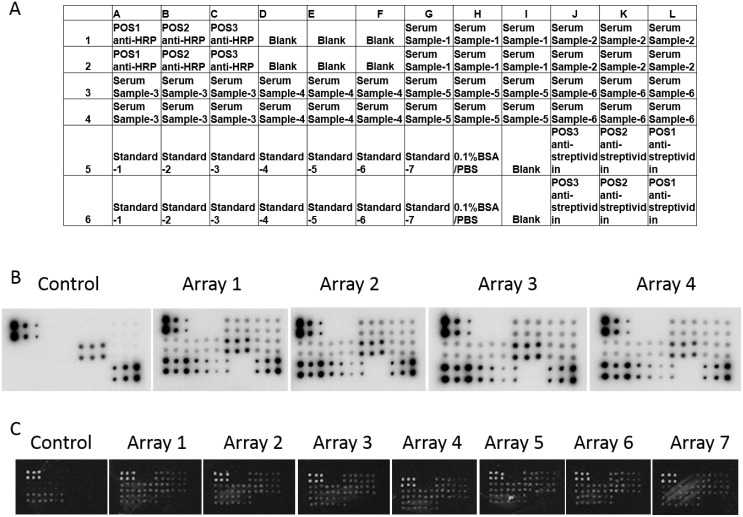
Determination of the solid support matrix for the RPPA system to detect targets in serum samples (**A**) The print map of RPPA array. (**B**) RPPA assay of MMP-9 in 6 serum samples on NC membranes. Arrays were printed according to the map. (B) Array 1 to Array 4 indicate 4 parallel experiments performed separately. Incubation without anti-MMP-9 antibody was used as a negative control. (**C**) RPPA assay of MMP-9 in 6 serum samples on glass slides. Array 1 to Array 7 indicate parallel experiments performed separately. Incubation without anti-MMP-9 antibody was used as a negative control.

First, two widely applied solid support matrices in protein arrays, nitro-cellulose (NC) membranes and glass slides, were tested to investigate the appropriateness for the RPPA. Serum samples at a 40-fold dilution were printed in groups of 6 onto nitro-cellulose membranes or glass slides as designated (Figure 1A) to generate arrays. Positive controls and serially diluted standard antigens were also printed on the support matrices as designated and used for quantitative analysis. After probing with an anti-MMP-9 antibody on 6 membranes and 7 glass slides, MMP-9 protein expression in six serum samples was determined (only 4 membranes shown in Figure 1). As shown in Figure 1B and 1C, signal intensity and spot size in both the samples and standards on NC membranes was more similar within and between arrays compared to those on glass slides. Quantitative analysis (Table 1) indicates that for the observed concentration of MMP-9 on NC membranes, the intra-assay CV ranged from 3.03 to 7.15% for the six serum samples and the inter-assay CV from the 6 assays ranged from 2.39–6.34%. However, the intra-assay and inter-assay CV of the 7 glass slide assays were significantly higher, ranging 21.37–40.19% and 16.33–131.47%, respectively for the 6 serum samples. Collectively, these results suggest that the NC membrane has advantages as a solid support matrix in stability and repeatability for RPPA when assaying serum samples.

**Table 1:** Comparison of precision of RPPA system on NC membrane and glass slides

Target Type	Arrays on NC membranes				Arrays on glass slides	
	MMP-9				MMP-9	
	Intra-assay precision			Inter-assay precision	Intra-assay precision	Inter-assay precision
**Repeat times (*n*)**	2	4	6	6	6	7
**Mean (ng/ml 6 samples)**	49.51–83.05	48.82–84.71	48.53–81.75	41.54–83.28	122.68–895.87	118.18–844.12
**Standard Derivation (6 samples)**	2.24–5.51	1.998–5.99	1.665–6.30	0.9929–5.28	27.13–327.53	56.06–248.60
**CV (%, 6 samples)**	2.69–5.52	3.65–5.07	3.03–7.15	2.39–6.34	21.37–40.19	16.33–131.47

**Figure 2:**
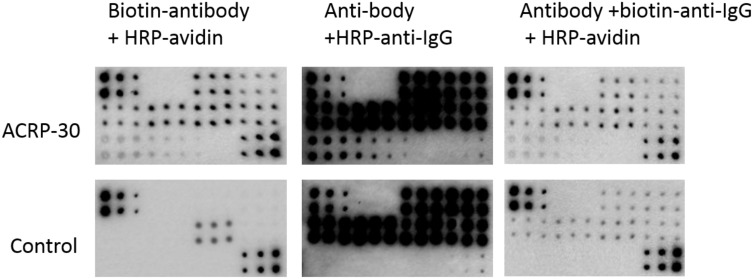
RPPA detection system tested with different antibody labeling options Serum samples and serially diluted standard antigen were printed directly on NC membranes. Protein expression was determined with the corresponding antibody with different labeling combinations and ECL detection. ACRP30 in serum samples and serially diluted pure standard with a concentration from 2000 ng/ml to 31.25 ng/ml was detected with a biotin-labeled anti-acrp30 antibody and HRP-labeled avidin (top left panel), or an anti-acrp30 antibody with a HRP-labeled Anti-IgG antibody (top middle panel), or an anti-acrp30 antibody with a biotin-labeled Anti-IgG and a HRP-labeled avidin (top right panel). The respective controls use the same antibody combination but without the anti-acrp30 antibody (bottom panels).

Next, we tested different antibody labeling combinations with the same ECL colorimetric assay to detect ACRP30, MMP-9 and hVEGF in serum samples on NC membranes, as examples of protein detection. To optimize the signal output, three combinations in the RPPA reaction were explored, including 1). a biotin-labeled anti-Acrp30 primary antibody with a HRP-labeled avidin secondary antibody; 2). an anti-Acrp30 primary antibody with a HRP-labeled anti-IgG secondary antibody; and 3). an anti-Acrp30 primary antibody with a biotin-labeled anti-IgG secondary antibody and HRP-labeled avidin. Arrays without primary antibody incubation were used as controls. Using the same stain and same concentration of primary antibody, the detection of Acrp30 in serum samples (shown in Figure 2) indicated that the strongest signals were displayed with combination 2) above; however, both combinations 2) and 3) resulted in high nonspecific signals in both target assay and controls. In contrast, combination 1) produced a high intensity signal but very low background in controls. Quantitative results of the signal from detected targets (Table 2) showed that the antibody combination 1) resulted in the highest sensitivity with a detected minimum concentration of 31.25, 31.25, and 62.5 ng/ml in standard antigens ACRP30, MMP-9 and hVEGF, respectively and efficient concentration of these targets in serum were obtained successfully in this antibody combination but not in combinations 2) and 3) because of the high levels of nonspecific signals and background. These results demonstrate that the combination of a biotin-labeled primary antibody with a HRP-labeled avidin secondary antibody in the RPPA system for serum sample detection was the best.

**Table 2:** Sensitivity of different detection methods and concentration of detected targets in serum samples

	Biotin-antibody + HRP-avidin			Anti-body + HRP-anti-IgG			Anti-body + biotin-anti-IgG + HRP-avidin		
	ACRP30	MMP-9	VEGF	ACRP30	MMP-9	VEGF	ACRP30	MMP-9	VEGF
**Detected minimum concentration (ng/ml)**	31.25	31.25	62.5	0.0625	0.125	0.25	0.25	0.25	0.25
**FRU**	688.0435	3533.214	256	5721.026	1571.579	3302.172	824.1898	266.2696	1914
**SD**	310.1039	151.2993	48.08352	1937.839	114.9565	628.8396	429.2707	179.6719	83.4386
**Detected serum concentration (ng/ml, *n* = 6)**	3965.07 ± 898.70	77.27 ± 29.25	0.49680 ± 0.21049	–	–	–	–	–	–

- Undetectable because of high background.

**Figure 3:**
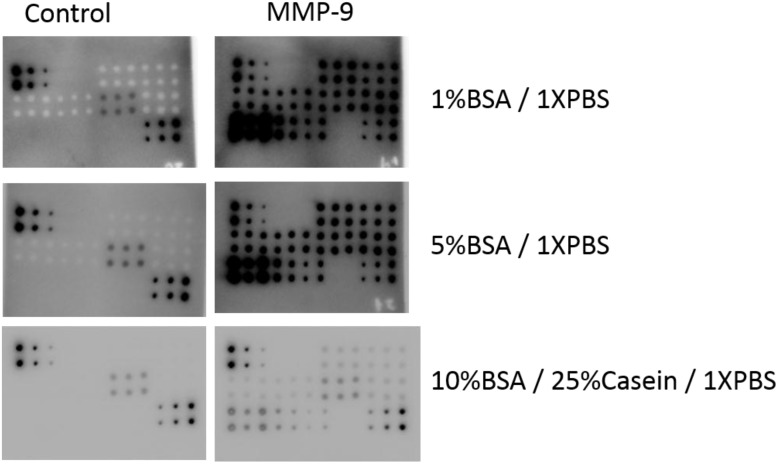
RPPA detection system tested with different blocking buffers Serum samples and serially diluted MMP-9 standard antigen were printed directly on NC membranes. Protein expression was determined with a biotin-labeled anti-MMP-9 antibody with HRP-avidin and ECL detection. 1%BSA/1XPBS (top panel), 5%BSA/1XPBS (middle panel), and 10%BSA /25%Casein/ 1XPBS (bottom panel) buffers are tested as blocking buffers. The same array and detection system without the anti-MMP-9 antibody were used as controls (left panels, respectively).

To optimize the minimum amount of background, different blocking buffers including 1X PBS containing 1% BSA, 1X PBS containing 5%BSA, or 1X PBS containing 10% BSA with 25% Casein were compared in the RPPA to detect MMP-9 from serum on NC membranes. As shown in Figure 3, maintaining all the other reaction conditions the same, the RPPA assay with 1X PBS/1% BSA and /5%BSA blocking buffers resulted in a high intensity of signals but also extremely high background. In contrast, blocking the arrays with 1X PBS containing 10% BSA and 25% Casein resulted in a moderate signal intensity with low background in both target assays and controls. This blocking buffer has also resulted in low background in other target assays with different arrays including antibody arrays (data not shown). Therefore, 1X PBS containing 10% BSA and 25% Casein was chosen as the optimized blocking buffer for this serum RPPA assay.

**Figure 4:**
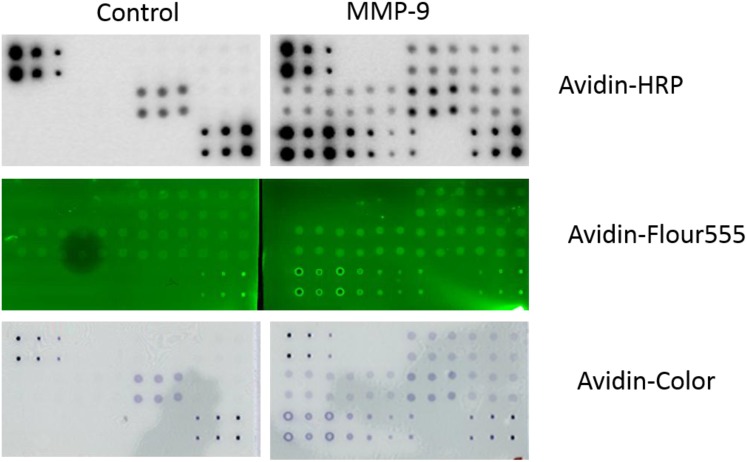
RPPA detection system tested with different imaging agents Serum samples and serially diluted MMP-9 standard antigen were printed directly on NC membranes. Protein expression was determined with a biotin-labeled anti-MMP-9 antibody with a HRP-avidin for ECL detection (top panel), an Alexa Flour555-avidin for laser scanning at 532 nm wavelength (middle panel), and a HRP-avidin for DAB detection (bottom panel). The same array and same antibody combinations without the anti-MMP-9 antibody were used as controls (left panels, respectively).

To establish the optimal signal reporting system for the RPPA that can detect targets in serum samples, avidin conjugated with Alexa Flour555 or HRP, which allowed for a direct fluorescent readout or detection via chemiluminescence or dye precipitation (DAB), respectively, were investigated using detection of MMP-9 as an example. Membranes incubated with the avidin-conjugated complex with the biotin-labeled capture antibody-antigens were visualized with a laser scanner or CCD camera exposure as shown in Figure 4 and Table 3. Even though direct scanning of Alexa Flour555 fluorophore provided the highest target signals, it also caused high background on the NC membranes, resulting in a lower detection sensitivity of the standard antigen. In contrast to both the Alexa Fluor555 and DAB color imaging, the highest sensitivity and lowest background were obtained from the signal reporting of chemiluminescence imaging.

**Table 3:** Comparison of different method for signal reporting in RPPA system

	Avidin-HRP	Avidin-Flour555	Avidin-Color
**Target**	MMP-9	MMP-9	MMP-9
**Detected minimum concentration (ug/ml)**	0.03125	0.0625	0.125
**FRU**	3461	5184.283	1429.4848
**SD**	74.24621	1045.8583	103.4423
**Detected serum concentration (ng/ml, *n* = 6)**	59.390 ± 17.393	2.89 ± 12.925	0.995353 ± 0.59073

Collectively, using NC membranes as the support matrix, a biotin-labeled primary antibody plus a HRP-labeled avidin secondary antibody, chemiluminescence as the signal detection system, and 1X PBS containing 10% BSA and 25% Casein as the blocking buffer optimized the intravariability and intervariability of MMP-9 expression of 2–6 duplicate spots in the same array membrane across 6 different membranes in serum samples with the RPPA system (Figure 1 and Table 1). The coefficient of variation (CV, SD divided by the average) was between 2.39 and 7.15%, less than 10%, suggesting the reliability of our established system.

### Quantification of proteins in serum samples using the RPPA assay

**Figure 5:**
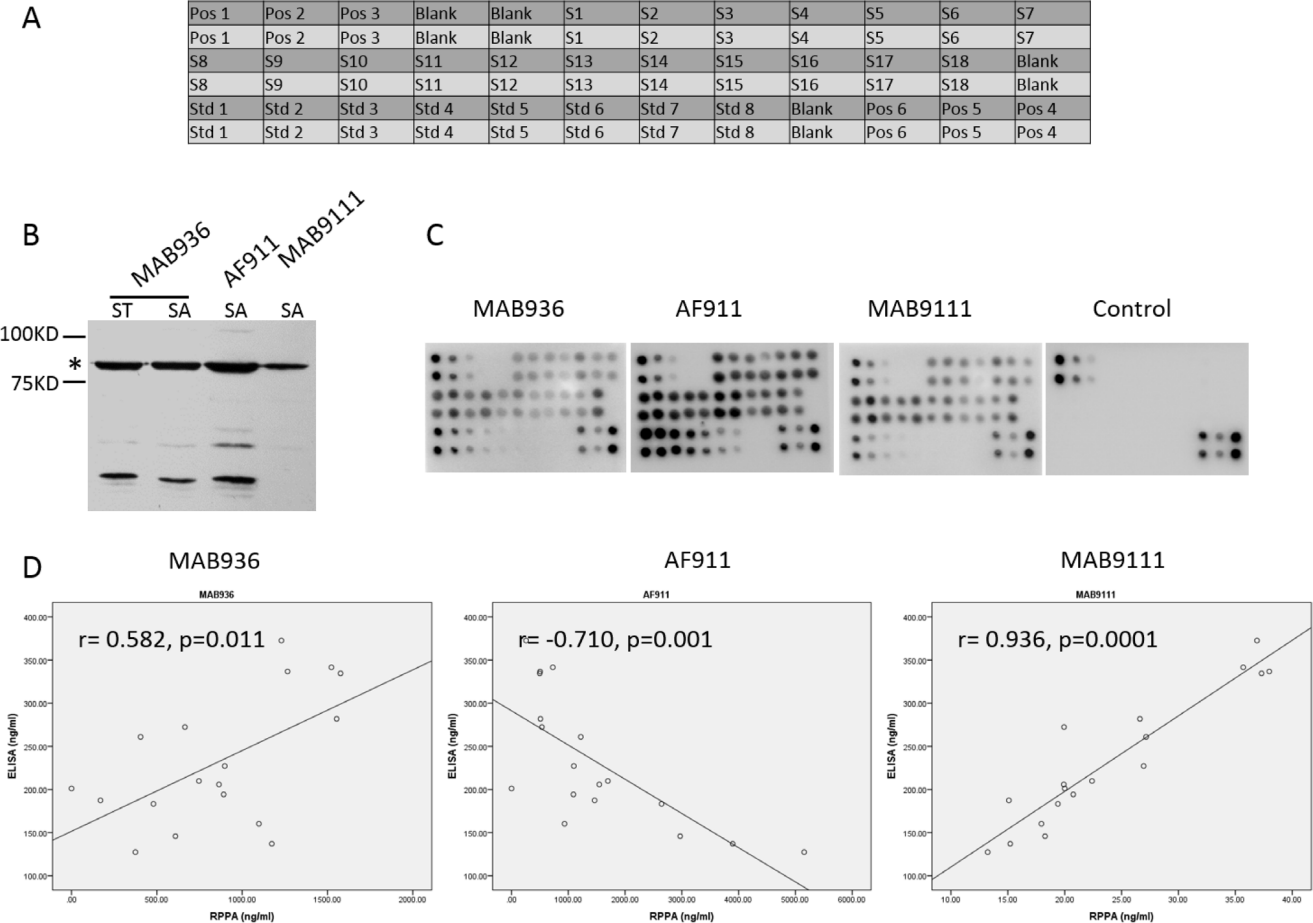
Detection of MMP-9 in 18 serum samples by established RPPA system and investigation of the quality of antibodies employed in RPPA showing different correlation of RPPA and ELISA (**A**) The map of RPPA array for serum samples detection. (**B**) Western blotting of serum sample (SA) and purified MMP-9 (ST) probed by antibodies MAB936, AF911, and MAB9111 respectively. ^*^indicates the band location of MMP-9 (around 84Kd). (**C**) Results of MMP-9 detected by RPPA method in 18 serum samples using MAB936, AF911, and MAB9111 as primary antibody respectively. (**D**) Correlation of RPPA with ELISA assay in MMP-9 detection in identical18 serum samples analyzed by Pearson Correlation in SPSS.

Using the established RPPA assay system described above, detection of MMP-9 in additional serum samples was done to validate the efficiency and accuracy of the assay system. Arrays were made as shown in Figure 5A. 18 serum samples, serially diluted standard antigens and controls were included. Membranes were then incubated with the biotin-labeled anti-MMP-9 antibody, the HRP-Streptavidin, and ECL substrates. MMP-9 expression in the 18 serum samples and standard antigens is shown in Figure 5C and the concentration of MMP-9 in each sample was calculated based on the standard curves derived from the serially diluted standard antigens (Table 4).

**Table 4:** Concentration of MMP-9 detected by ELISA with commercial kit and RPPA assay with corresponding antibody

MMP-9	ELISA (ng/ml)	RPPA assay (ng/ml)		
		MAB9111	AF911	MAB936
**Serum 1**	281.81	26.61	509.36	1553.63
**Serum 2**	187.34	15.09	1463.71	169.69
**Serum 3**	160.20	17.95	934.36	1098.21
**Serum 4**	194.23	20.75	1090.33	891.64
**Serum 5**	137.08	15.21	3895.77	1173.73
**Serum 6**	201.16	20.00	0.00	0.00
**Serum 7**	209.80	22.40	1697.66	746.62
**Serum 8**	334.56	37.30	496.50	1576.51
**Serum 9**	341.58	35.69	727.54	1522.98
**Serum 10**	272.33	19.94	535.20	664.56
**Serum 11**	260.90	27.15	1220.34	404.88
**Serum 12**	227.17	26.94	1097.50	897.86
**Serum 13**	372.61	36.90	256.46	1229.85
**Serum 14**	336.74	37.98	502.92	1266.10
**Serum 15**	145.81	18.27	2970.56	609.03
**Serum 16**	183.26	19.40	2643.36	480.14
**Serum 17**	205.81	19.92	1546.16	864.02
**Serum 18**	127.42	13.23	5154.74	374.48

To evaluate the quality of antibody used for this RPPA assay, 3 different anti-MMP-9 antibodies including MAB936, MAB9111 and AF911 (R&D, Minneapolis, MN) were tested. Western blotting of serum samples with these antibodies and the same HRP-anti-IgG secondary antibody showed that MAB9111 resulted in single band, but both of the other two antibodies showed multiple bands in the same serum sample (Figure 5B). Correspondingly, the RPPA assay conducted with these 3 antibodies for the 18 serum samples also displayed different signal intensity (Figure 5C) and MMP-9 concentration (Table 4). To validate the accuracy of the RPPA assay, the concentration of MMP-9 determined by RPPA with the 3 antibodies was compared with the MMP-9 concentrations determined using a commercial ELISA kit. As shown in Figure 5D, correlation of MMP-9 concentrations of the RPPA assay with the ELISA assay demonstrated the most accurate detection of MMP-9 in serum samples was obtained using the MAB9111 antibody in the RPPA detection system with *r* = 0.936, *p* = 0.0001 vs. *r* = 0.582, *p* = 0.011 with MAB936 and *r* = −0.71, *p* = 0.001 with AF911. Other targets, such as Acrp30 and hVEGF, detected by both RPPA and ELISA also displayed good correlation coefficients between RPPA and ELISA when antibodies with a single band in western blot were used in the RPPA system (Table 5). Taken together, these results strongly suggest the necessity of antibody validation by western blot, where presentation of a single band should be the first step to ensure success in the RPPA system for serum protein detection.

**Table 5:** Validation of antibodies used for RPPA by western-blot (W-B) and ELISA

Target	antibody	W-B (pattern of bands)	ELISA (average concentration, *n* = 40)	RPPA (average concentration, *n* = 40)	Correlation coefficient between RPPA and ELISA (R,P)
**MMP-9**	MAB9111	S	232.22 ng/ml	24.92 ng/ml	*R* = 0.936, *P* < 0.0001^*^
	AF911	M	232.22 ng/ml	1485.69 ng/ml	*R* = –0.71, *P* = 0.001
	MAB936	M	232.22 ng/ml	862.44 ng/ml	*R* = 0.582, *P* = 0.011
**Acrp30**	MAB10651	M	89.85 ng/ml	723.83 ng/ml	*R* = 0.39, *P* = 0.401
	BAM1065	M	89.85 ng/ml	2.64 ng/ml	*R* = 0.504, *P* = 0.092
	AF1065	S	89.85 ng/ml	3144.45 ng/ml	*R* = 0.971, *P* < 0.0001^*^
**hVEGF**	PB0276B	M	0.42 ng/ml	382.37 ng/ml	*R* = 0.346, *P* = 0.533
	MAB293	M	0.42 ng/ml	0.47 ng/ml	*R* = 0.691, *P* = 0.067
	BAF293	M	0.42 ng/ml	1184.23 ng/ml	*R* = –0.62, *P* = 0.054
**ApoA**		S	114.07 ug/ml	1235.67 ug/ml	*R* = 0.989, *P* < 0.0001^*^
**ApoE**		S	54.97 ug/ml	20.59 ug/ml	*R* = 0.942, *P* < 0.0001^*^
**A2M**		S	707.94 ng/ml	4.17 ng/ml	*R* = 0.746, *P* = 0.0002^*^
**Clusterin**		S	117.83	58.22	*R* = 0.176, *P* = 0.278
**CRP**		S	0.55	0.32	*R* = 0.769, *P* = 0.0003^*^

S: single band from western-blot; M: multiple bands from western-blot; *P* < 0.05 means statistically significant; ^*^means good correlation of RPPA with ELISA.

**Figure 6:**
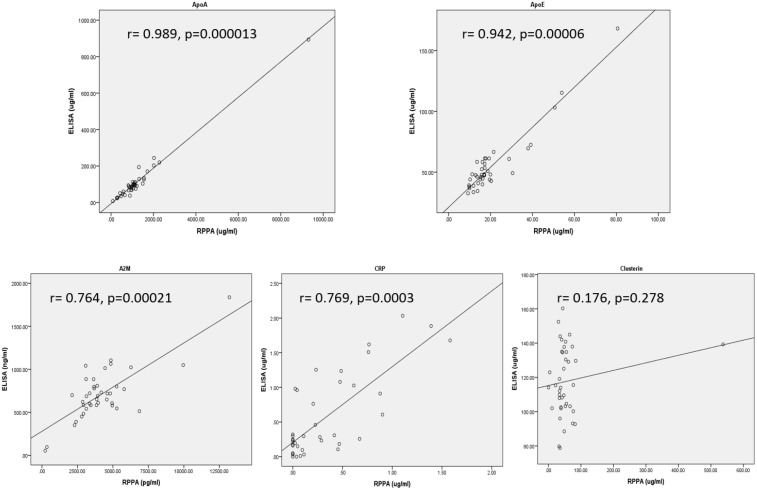
Correlation of RPPA with ELISA with detection of ApoA (top left panel), ApoE (top right panel), A2M (bottom left panel), CRP (bottom middle panel), and Clusterin (bottom right panel) from 40 serum samples ELISA were performed with commercial kits and RPPA assays used western blot validated antibodies. Correlation was analyzed by Pearson Correlation in SPSS.

To further determine the suitability of particular antibodies in the RPPA assay, more targets including Apo-E, Clusterin, CRP, A2M and Apo-A were assayed by RPPA in 40 serum samples with western blot validated antibodies, and the accuracy was evaluated by correlation analysis with ELISA. As shown in Figure 6 and Table 5, even though each antibody had been tested by western blot to exclude nonspecific reactions as indicated by the presence of multiple bands (data not shown), correlation of RPPA with ELISA varied between detected targets with *r* values ranging from 0.989 to 0.176. The detection of ApoA and ApoE by RPPA displayed excellent correlation (*r* = 0.989 and 0.942); however, RPPA assay of Clusterin showed no correlation with ELISA (*r* = 0.176, *p* = 0.278). These data suggest the importance of validation by different assay platforms for RPPA, and that using only western blot to test antibody quality is not stringent enough.

### Evaluation of biomarkers of HCC in serum samples

**Figure 7:**
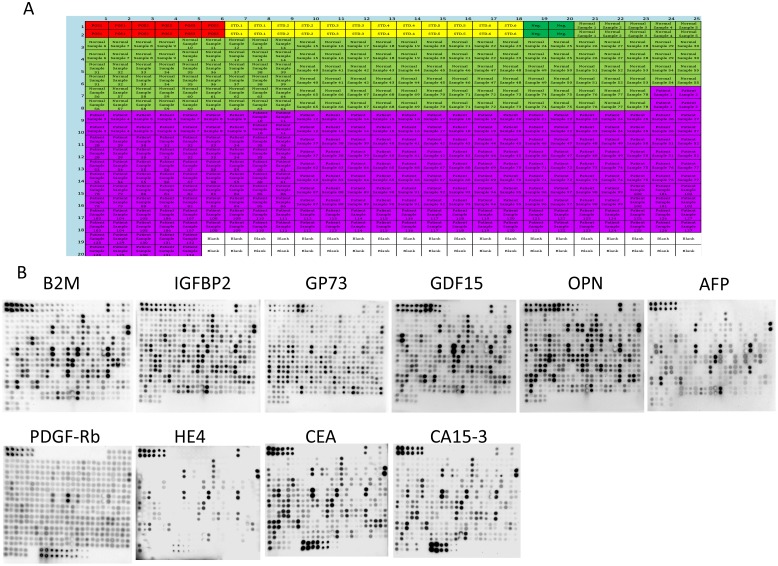

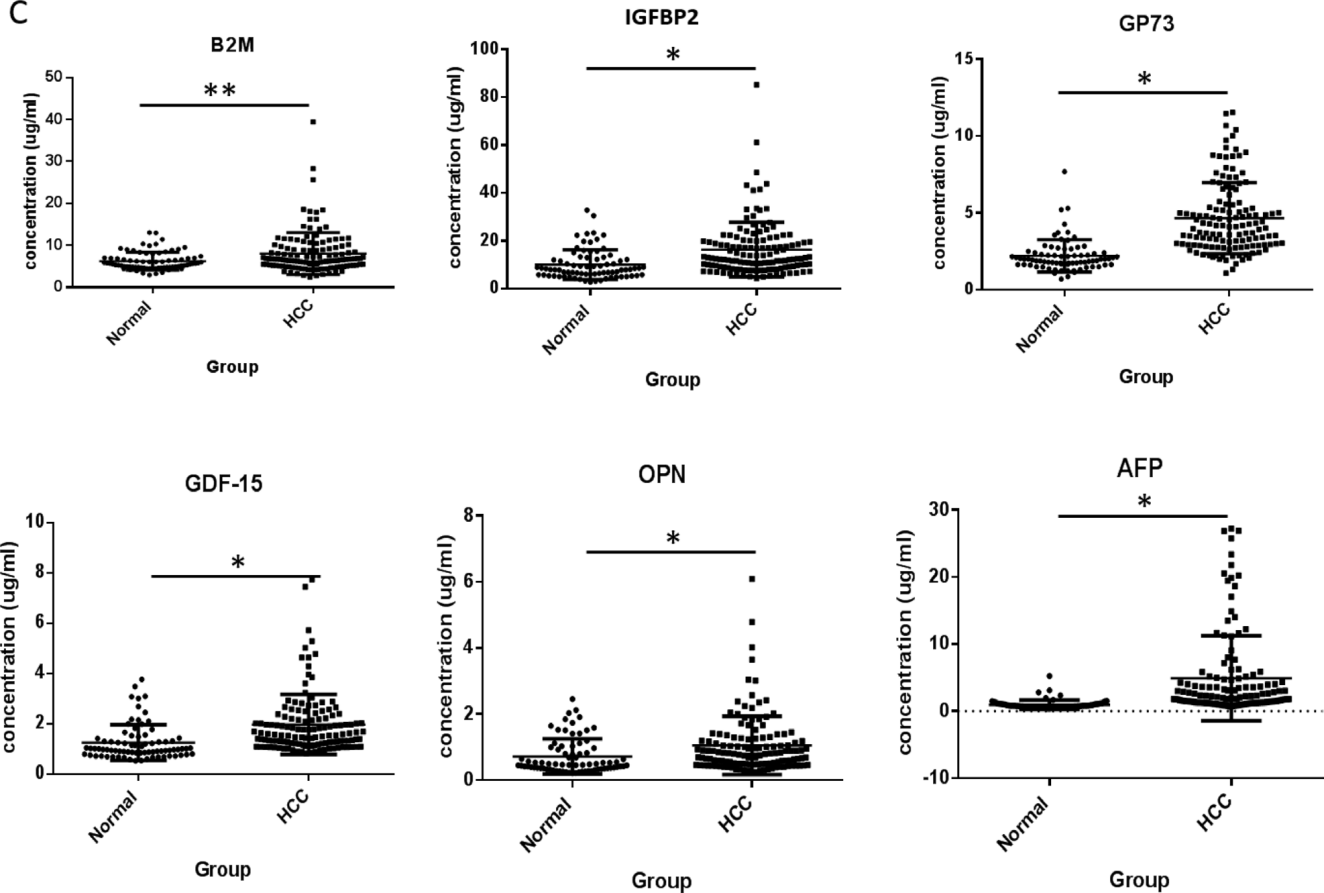

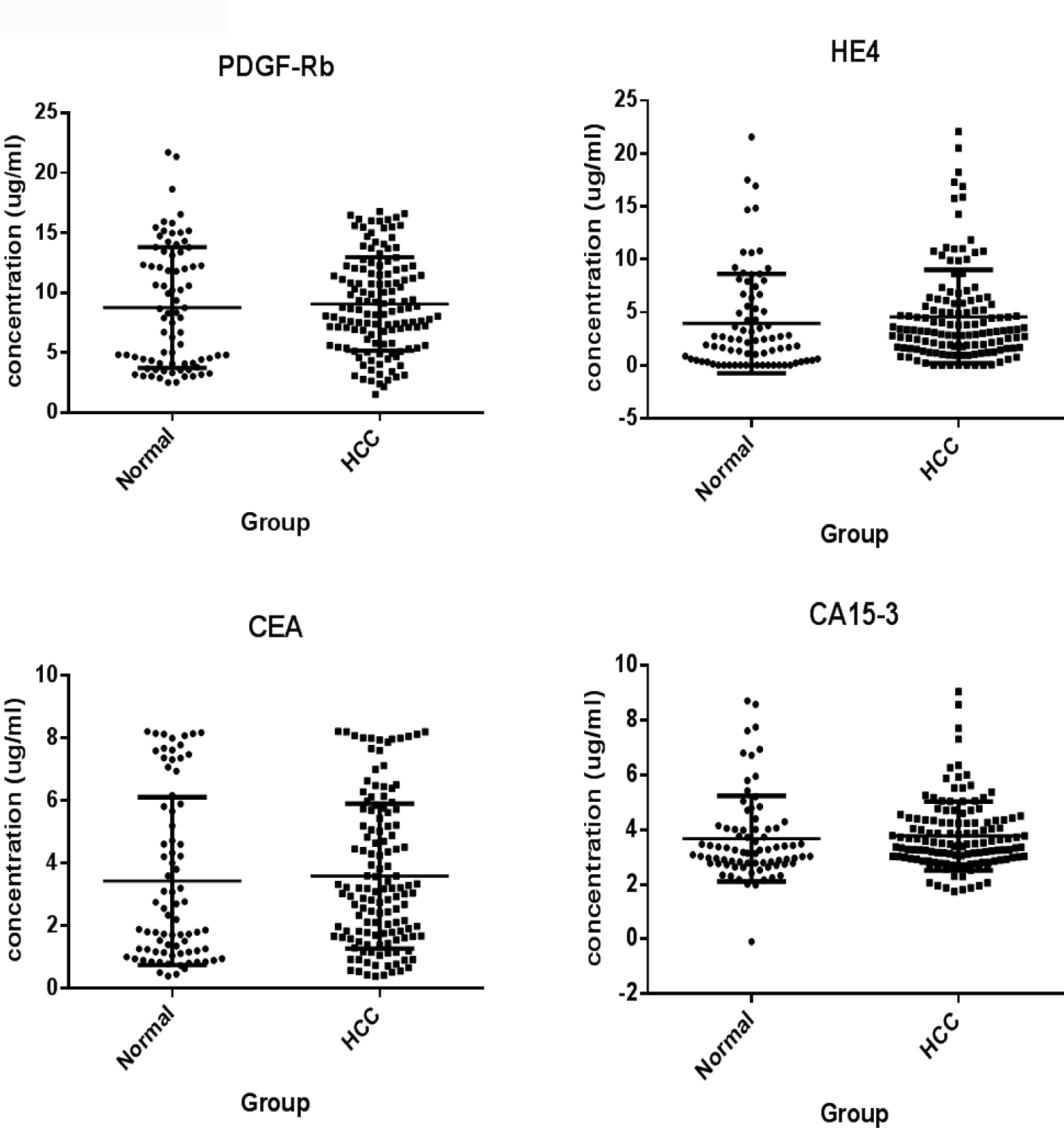
RPPA analysis of 10 targets AFP, B2M, CA15-3, CEA, HE4, IGFBP2, GP73, GDF15, OPN and PDGF-Rb in serum of HCC patients for potential of clinical diagnosis 132 serum samples from HCC patients and 78 serum samples from normal controls were detected with RPPA. (**A**) Array map for printing with serum samples, standards, and controls. (**B**) Results of RPPA assay for each detected target on NC membranes. (**C**) Quantitative and statistical analyses of each detected target, ^*^P < 0.001. ^**^P < 0.005

Using the optimized parameters determined above in the established RPPA system, we quantitatively measured the expression levels of 10 proteins including AFP, B2M, CA15-3, CEA, GDF-15, GP73, HE4, IGFBP-2, OPN and PDGF-Rb in serum samples of 132 hepatocellular carcinoma (HCC) patients and 78 healthy controls. HCC patients were classified by clinical standards according to Asian-Pacific Association for the Study of Liver (APASL) guide lines. Normal serum sample were from healthy volunteers. The study was approved by the Committees for Ethical Review of Research Involving Human Subjects at Sun Yat-sen University. For each protein target, the antibody used was first qualified for the RPPA assay by evaluation with western blot and correlation analysis with ELISA as described above. All samples were spotted on the membranes according to the map shown in Figure 7A and then the assays were performed with the corresponding antibodies. The signals from each sample and the corresponding detected targets are shown in Figure 7B. Quantitative analysis of expression levels of the 10 proteins is shown in Figure 7C and Table 6. The average expression of AFP and GP73 in 132 HCC serum samples showed a dramatic increase (5.16 and 2.12-fold change) compared with the levels in healthy controls (mean concentration of 4.904 vs. 0.9404 μg/ml and 4.644 vs. 2.192 μg/ml, respectively). The expression levels of 4 proteins B2M, GDF-15, IGFBP-2, OPN has also been determined for HCC patients (mean concentration of 8.024, 1.972, 16.367, 1.052 μg/ml) and Normal controls (6.231, 1.266, 10.106, 0.714 μg/ml), and the fold change differences ranged from 1.29 to 1.62. The average expression of another 4 proteins CA15-3, CEA, HE4 and PDGF-Rb, however, showed no difference between two groups with fold change of 1.02, 1.04, 1.16 and 1.04 respectively. Statistical analyses determined significant differences between the HCC and normal groups for 6 proteins including AFP, GP73, B2M, GDF-15, IGFBP-2 and OPN but not for CA15-3, CEA, HE4 and PDGF-Rb (Table 6 and Figure 7C).

**Table 6:** Results of 10 targets detected by RPPA in HCC and Normal serum samples

Protein ID	Mean_HCC (μg/ml, *n* = 132)	Mean_Normal (μg/ml, *n* = 78)	Fold of Change	*P*. Value (Mann–Whitney test)
**AFP**	4.9 ± 6.33	0.95 ± 0.7	5.16	<0.0001
**B2M**	8.02 ± 5	6.23 ± 2.14	1.29	0.0045
**CA15-3**	3.77 ± 1.26	3.68 ± 1.56	1.02	0.1257
**CEA**	3.59 ± 2.32	3.43 ± 2.68	1.04	0.2658
**HE4**	4.6 ± 4.42	3.96 ± 4.69	1.16	0.1207
**IGFBP2**	16.37 ± 11.4	10.11 ± 6.22	1.62	<0.0001
**GDF15**	1.97 ± 1.2	1.25 ± 0.71	1.58	<0.0001
**GP73**	4.64 ± 2.31	2.19 ± 1.05	2.12	<0.0001
**OPN**	1.05 ± 0.88	0.71 ± 0.53	1.48	<0.0001
**PDGF-Rb**	9.08 ± 3.9	8.69 ± 5.19	1.04	0.3207

**Figure 8:**
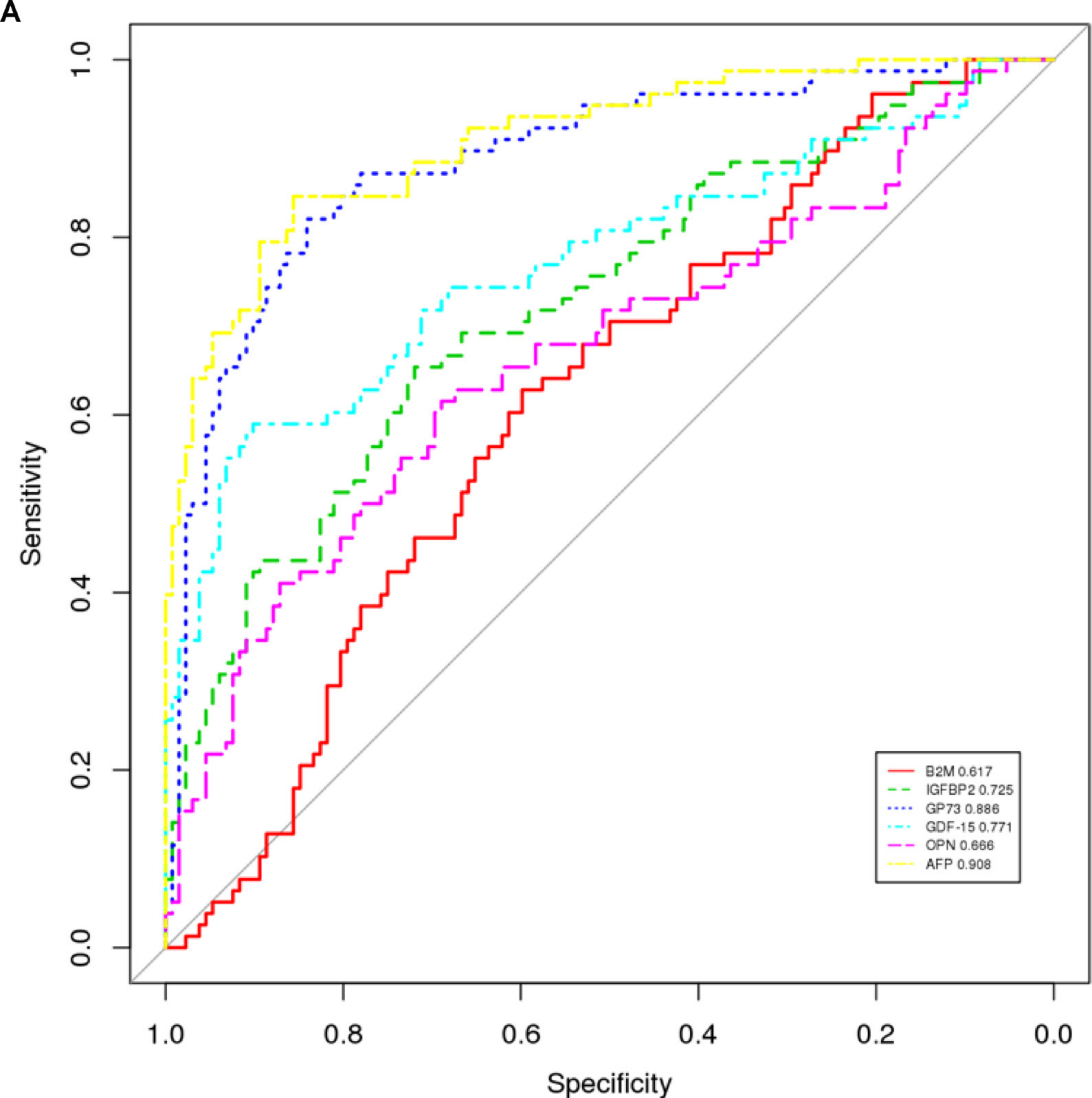

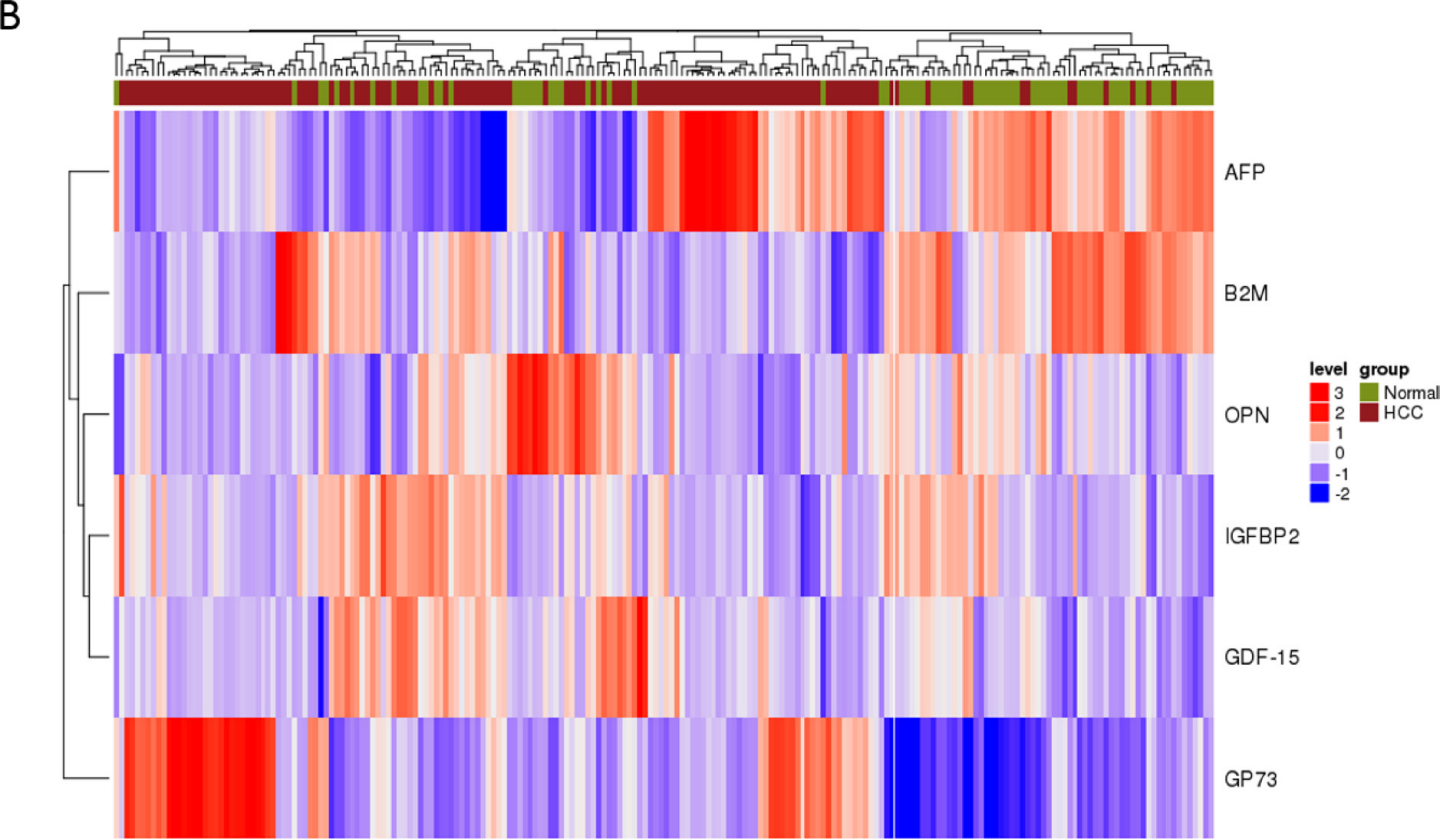

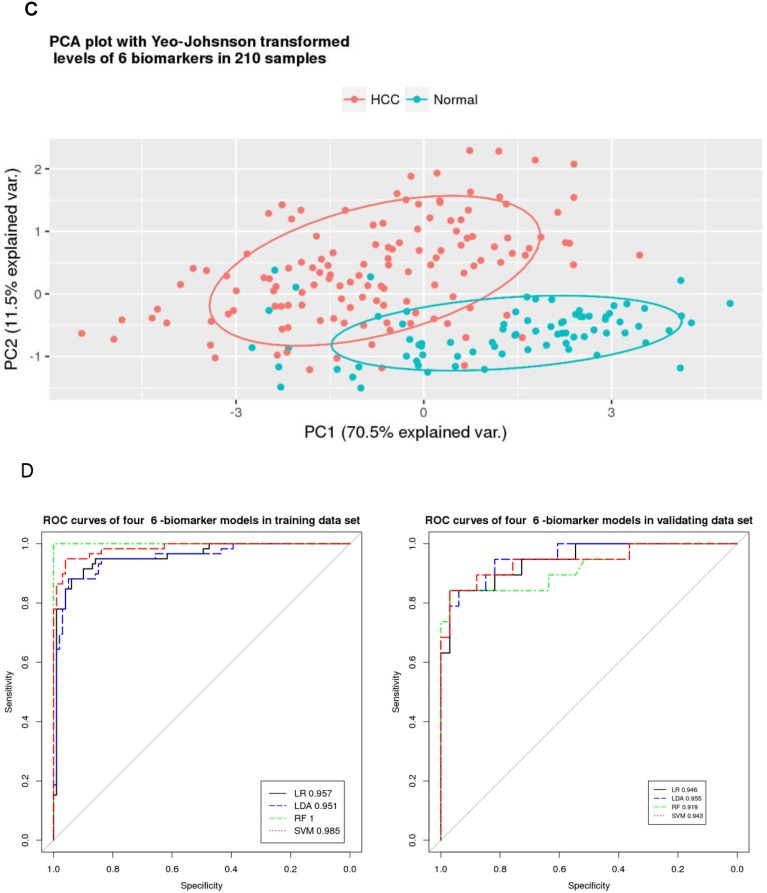
Classification analysis of 6 RPPA-measured proteins in 210 serum samples (132 HCC patients and 78 healthy controls) to evaluate their diagnostic performance as biomarkers of HCC (**A**) The individual ROC curve for each biomarker. (**B**) Hierarchic clustering of HCC and healthy samples based on expression level changes of all 6 biomarkers. (**C**) Evaluation of diagnostic performance of all 6 biomarkers against HCC patients (red) and healthy controls (blue) with PCA analysis. (**D**) AUC rate of the combined 6 biomarkers (B2M, IGFBP2, GP73, GDF15, OPN and AFP) as a specific HCC signature evaluated by supervised LR, LDA, RF and SVM models in a training set and a validation set as described in the Methods section.

To further evaluate the potential role of these 6 proteins as biomarkers of HCC, additional statistic and bioinformatic analyses were conducted based on the RPPA results. First, ROC curves were generated for the 6 proteins (Figure 8A) displaying different accuracy ranging from 0.617 (B2M) to 0.908 (AFP), which indicated the capacity of each protein to distinguish the HCC patients from healthy controls. Second, principal component analysis (PCA) showed that the expression levels of these 6 proteins could successfully separate HCC patients from healthy controls (Figure 8B). Additionally, correlation heatmap analysis yielded separate clusters that consisted of mostly HCC patients and healthy controls respectively, determining clear differences between the two groups (Figure 8C). To find out the optimal HCC specific signature to distinguish HCC from healthy samples, different combinations of the 6 proteins were analyzed with several prediction models, including a linear discriminant algorithm called linear discriminate analysis (LDA), a logistic regression (LR), a random forest (RF) and a support vector machine model (SVM). The 132 HCC and 78 healthy samples were divided into a training set and a validation set as described in the Methods section. Starting with a combination of AFP and GP73, the 2 proteins which individually offered the best ROC in the above analysis, different combinations of 2, 3, 4, 5, and 6 proteins were tested and the accuracy, specificity and sensitivity of each combination was assessed (data not shown). The combination of all 6 proteins: AFP, B2M, GP73, GDF15, IGFBP2 and OPN displayed the best results in the 4 models tested in both the training and testing set in distinguishing HCC patients from healthy controls, with the highest accuracy rate of 0.923 (Figure 8D). Taken together, these comprehensive analyses indicate that the 6 proteins detected by RPPA in serum samples have the potential to be diagnostic biomarkers of HCC. This study demonstrates a simplified and robust, high-throughput technology that can be used for the quantification of proteins in serum. This technology can be applied to biomarker screening in serum samples.

## DISCUSSION

In RPPA, thousands of individual samples are immobilized on a solid support matrix by an arrayer so that the arrayed samples can be recognized simultaneously with highly specific antibodies against desired targets. The protein-antibody complexes are then visualized by a signal reporting system such as fluorescence or chemiluminescence to quantify the expression of the assayed proteins. The advantages of RPPA are manifest in several aspects, such as being high-throughput, cost efficiency, requiring minimal sample consumption, and being easy to manipulate [14]. To promote the application of RPPA technology in the detection of protein targets in serum samples and to make RPPA a feasible technology for the discovery and validation of blood-based biomarkers, we have developed and optimized an RPPA system specifically for serum samples based on our previous study. In this study, we concluded that the optimized RPPA system consisted of the following: 1). a NC membrane as array support matrix; 2). the combination of a biotin-labeled primary antibody plus an enzyme-conjugated avidin secondary antibody for target detection; 3). signal detection via the chemiluminescence method; 4). 1X PBS containing 10% BSA and 25% Casein as the blocking buffer to minimize background; and more importantly, 5). the validation of each antibody for RPPA through two platforms of both western blot and ELISA. Applying this optimized RPPA system, we tested a total of 210 serum samples from HCC patients and healthy volunteers (132 vs. 78, respectively). The expression levels of 6 proteins were determined, and were able to statistically distinguish the patients from the healthy controls. Our results suggest that the optimized RPPA system can potentially be a very powerful tool for biomarker discovery in serum samples.

Nitrocellulose membranes or glass slides are commonly used as solid support matrices for arrays such as DNA arrays and antibody arrays [8, 15]. Nitrocellulose-coated slides or membranes have superior protein binding and better protection of tertiary protein structures, and therefore, improved stability of protein interactions compared to glass slides [16, 17]. Glass slides pre-coated with chemical moieties, however, are more compatible with fluorescence detection and have better background, and are widely used for quantitatively analysis of protein targets [18–20]. To determine the optimal support matrix specifically for serum sample detection with an RPPA assay, we tested both NC membranes and aminosilane-coated glass slides printed with different dilutions of serum. Despite the high intensity of signals derived from fluorescence detection, glass slides resulted in huge inter- and intra-assay CV which was unacceptable for quantitative analysis, whereas NC membranes offered obvious advantages of great stability with less than 10% inter- and intra-assay CV (Figure 1 and Table 1). Aminosilane-coated glass slides have been successfully adopted for printing proteins in buffers for quantitative assessment [21, 22]. The large variation of detection observed here was probably caused by the high viscosity of serum samples which bind poorly on glass slides even when samples are diluted one hundred-fold. Thus, we chose the NC membrane as the best solid support matrix for RPPA for assaying serum samples. Fluorescence dye or enzyme conjugated anti-IgG secondary antibodies are commonly employed to facilitate and amplify signal detection in RPPA assays for detection of proteins from tissue or cell lysates [15]. However, our results indicate it is not suitable for serum sample detection since a large amount of IgG is present in serum, which produces a high background signal because of cross-reaction between species of the anti-IgG secondary antibody. The alternative approach of a biotin-labeled primary antibody along with a HRP-conjugated avidin secondary antibody not only dramatically reduced background signals but also provided a simpler procedure with a shortened assay (Figure 2 and Table 2).

For the signal detection system, a direct readout of fluorescence dye or detection via chemiluminescence have been currently used for protein array analysis. Fluorescence as the reporting signals has gained attention since the evolution of DNA microarrays due to its high dynamic range over several orders of magnitude that facilitate quantitative detection of target proteins with high sensitivity [23, 24]. However, the fluorescence reporting system requires a laser scanner that is expensive and not readily available. In addition, use of fluorescence on some support materials, such as NC membranes, produces high background signals and lower target signal intensity because of natural autofluorescence and scattering and reflecting of emission light [25]. Chemiluminescent signals can be monitored using X-ray film or CCD exposure systems available in most labs worldwide. Even though chemiluminescence reporting systems require appropriate exposure time and manipulation in case of signal fading, the use of systems controls have increased the accuracy for normalization and elevated the sensitivity through more delicate CCD instruments. In this study, we tested both a fluorescence detection system and a chemiluminescence system for our RPPA platform and found a much better signal to noise ratio for chemiluminescence system (ECL) compared with the fluorescence detection (Figure 4 and Table 3). The excellent CVs observed suggested the reliability of our established system.

Because only one antibody is employed to recognize the target from thousands of proteins that pile up in a micro dot, the quality of antibody is extremely important for the RPPA system. Therefore, the selection of the antibody is a much more important step for successful RPPA assays compared to traditional techniques like western blot and ELISA, which either separates proteins according to size on gels before probing with a single antibody or recognizes targets by a pair of antibodies to increase specificity of the assay, respectively. Many previous studies analyzing proteins in tissue samples or cell lysate by RPPA validated antibodies using western blot analysis and antibodies producing a single band against their targets were considered as good quality [15, 26, 27]. Antibodies showing more than a 0.7 *R* value of correlation between RPPA and western blot when detecting identical samples has been accepted [28]. In our RPPA system, antibodies targeted to the same protein were inspected by western blot with a serum sample, and only antibodies resulting in single band displayed good correlation of RPPA with ELISA in serum samples (Figure 5 and Table 5). Because serum is a more complicated matrix than cell lysate or fixed tissue samples, providing more opportunities for nonspecific interactions of antibodies–antigens, the single band selection by western blot was not stringent enough for our RPPA system for the detection of serum proteins. Specifically, some antibodies, such as anti-Clusterin, that showed a single band in western blot resulted in poor correlation of RPPA with ELISA (Figure 6). Thus, validation of antibodies by two platforms, including both western blot and ELISA, appears to be indispensable for RPPA detection of serum samples. This is consistent with a previous study by Grote *et al*. who investigated the presence of CA19-9 in serum and plasma by RPPA and compared it with ELISA [29]. Even though 300–400 antibodies to protein targets, including phosphorylated proteins which are important in a number of cancer related pathways, publicly available for RPPA to detect proteins in tissue and cell lysate have been validated and published [30], very few antibodies have been suitably validated for the RPPA system when investigating serum proteins. To make RPPA valuable for blood biomarker discovery and validation, additional future work is required.

In the application of our optimized RPPA system, we have detected 10 proteins, including AFP, B2M, CA15-3, CEA, GDF-15, GP73, HE4, IGFBP-2, OPN and PDGF-Rb in serum samples of 132 HCC patients and 78 healthy volunteers. AFP has been commonly used for clinical early diagnosis of liver cancer despite limited sensitivity and specificity [31, 32]. GP73 was previously reported to be significantly elevated in multiple tumors including lung adenocarcinoma [33], seminomas [34] and renal cell cancer [35], and recent studies have shown that a significantly elevated serum GP73 level is closely associated with liver diseases, particularly HCC [36, 37]. Consistent with these reports, the expression levels of AFP and GP73 detected by RPPA in our study were significantly upregulated in HCC patients compared with healthy controls and displayed the capability to distinguish between the two groups with high accuracy rates. These results strongly confirmed the reliability of our detection system. The other 6 proteins tested were chosen because of their reported involvement in liver diseases (e.g. GDF15, which has been shown to be involved in liver disease and hepatocellular carcinoma [38]) and other cancers (as is the case for CEA, IGFBP2, B2M, PDGF-Rb and OPN [39–42]). Our results showed that four of these 6 proteins displayed statistically significant differences in concentration between the HCC patients and healthy controls. CA15-3 and HE4 were FDA approved tumor biomarkers for breast cancer and ovarian cancer respectively [43]. The expression levels of these two proteins have been measured effectively, however, there was no different statistically between two groups, which again tested and confirmed the specificity and reliability of our our optimized RPPA technology for serum protein detection. We also investigated the best signature using multiple proteins as diagnostic biomarkers to distinguish these groups using predictive models. We determined that a combination of 6 proteins (AFP, B2M, GP73, GDF15, IGFBP2 and OPN) had the highest accuracy rate to distinguish HCC from healthy controls within samples detected in this study. While further studies are needed to investigate the potential biomarkers of HCC for diagnosis and/or prognosis, this study has not only demonstrated the feasibility and reliability but also revealed the advantages of RPPA as a robust technology to detect proteins in large numbers of serum samples rapidly for application in biomarker discovery.

## MATERIALS AND METHODS

### Materials

All antibodies were produced by our own or purchased from either BD PharMingen (San Diego, CA) or R&D (Minneapolis, MN). All cytokines were obtained from R&D (Minneapolis, MN). Horseradish peroxidase-conjugated streptavidin was purchased from BD PharMingen. Nitrocellulose membranes were purchased from Thermo Fisher (Waltham, MA). Amino-Silane coated glass slides were purchased from CORNING (Corning, NY). Purified antigens were stocked in stabilizer buffers and were serially diluted used as standards on the RPPAs.

### Sample preparation

Serum samples were collected from the third affiliated hospital, Sun Yat-Sen university. All serum samples were procured following standard operating procedures: whole blood was collected in BD vacutainer serum tubes, incubated undisturbed at room temperature for 30 min, and then centrifuged at 3000 rpm for 15 min at 4° C. The supernatant serum was divided into 200 μL and frozen at −80° C for storage. HCC patients were classified by clinical standards according to Asian-Pacific Association for the Study of Liver (APASL) guide lines. Normal serum sample were from healthy volunteers. The study was approved by the Committees for Ethical Review of Research Involving Human Subjects at Sun Yat-sen University.

### Array manufacture

Properly diluted serum samples (from 2 to 1000-fold) and serially diluted standard antigens were spotted onto nitrocellulose membranes or glass slides using a BioOdyssey Calligrapher MiniArrayer (Bio-Rad). Anti-HRP IgG and anti-Avidin IgG were used as positive controls and 1× PBS containing 1% BSA was used as a negative control. After printing, the slides and membranes were vacuum and naturally dried, respectively, and stored at −80° C until use.

### RPPA detection

After equilibration to room temperature, the glass slides or membranes were carefully removed from their packages, blocked with blocking buffer for 30 min at room temperature (RT), and then incubated with the corresponding target antibody combinations for 2 hrs. After extensive washing with TBS/0.1% Tween three times and TBS twice, the signals were visualized with a Genepix 4000B laser scanner (Molecular Devices, USA) at 532 nm for glass slides or with an Enhanced Chemiluminescence (ECL) system (ThermoFisher Scientific, USA) for membranes.

### ELISA assay

ELISA was performed according to the manufacturer's instructions (RayBiotech, Norcross, GA). The 96-well plates precoated with capture antibodies were blocked in 1% BSA/PBS for 1 hr at RT. After incubation with diluted serum samples and different concentrations of standard for 2 h, the plates were washed with TBS/0.1% Tween followed by 1 hr incubation of biotinylated detection antibody. After extensive washing, color development was done by incubation with substrate solution and the plate was read at 405 nm. Standard curves were generated with Sigmaplot and the concentrations of different samples were determined from the standard curves.

### Western blot

Serum proteins or purified standard antigens were separated by 10% SDS-PAGE and transferred to PVDF membranes (Bio-Rad, Hercules, CA, USA). Membranes were probed with individual primary antibodies followed by incubation of HRP-conjugated anti-mouse or anti-rabbit secondary antibodies. The signals were then visualized with the ECL Western Blotting Detection System (ThermoFisher Scientific, USA).

### Data analysis

The membrane signal intensities were analyzed using the LabWorks program (PerKin Elmer, Massachusetts, USA). Fluorescence units on glass slides were calculated with the GenePix Pro 7 program (Molecular Devices, USA). Standard curves were generated using Sigmaplot (Chicago, IL). Correlation analyses were performed by Pearson Correlation analysis using IBM SPSS Statistic 20 (SPSS Inc., Chicago, IL) and *p* < 0.05 was considered statistically significant.

### Classification analysis between HCC patients and healthy controls

Further classification analysis was performed for results from RPPA assay of 6 biomarkers with serum samples of 132 hepatocellular carcinoma (HCC) patients and 78 healthy controls. Receiver's operating characteristics curves (ROCs) were plotted for evaluation of diagnostic performance of biomarkers/methods. Primary Component Analysis and Hierarchic clustering analyses were implemented for clustering of subjects and biomarkers. Supervised linear discriminant analysis (LDA), logistic regression (LR), random forest (RF) and support vector machine (SVM) models were fitted with all 6 biomarkers against the diagnoses (Cancer/Control) for assessment of comprehensive diagnostic performance of the biomarker panel. All analyses were conducted with R 3.3.2 for linux. The model fitting of LR, SVM, LDA and RF was implemented with 3:1 sample-splitting, in which 3/4 of all the samples (99 HCC vs. 59 healthy controls) were randomly selected for model training and the remaining samples (33 HCC and 19 healthy controls) were used for validation/performance evaluation. A 4-fold cross-validation scheme was adopted during model training.
